# Estimating the prevalence of chronic infections among asymptomatic migrants: results of a screening programme in Catalonia, Spain

**DOI:** 10.1016/j.jmh.2024.100278

**Published:** 2024-10-30

**Authors:** Angeline Cruz, Angela Martínez-Perez, Alex Almuedo-Riera, Carme Roca Saumell, Marina Gigante Lopez, Oriol Gasch, Gemma Falcó, Ana Jiménez-Lozano, Consol Sanchez-Collado, Julio Alonso-Padilla, Juan Carlos Hurtado, Miriam J Álvarez-Martínez, Aina Casellas, Ana Requena-Méndez

**Affiliations:** a Barcelona Institute for Global Health (ISGlobal), Hospital Clínic-Universitat de Barcelona. Carrer Roselló 132, 08036 Barcelona, Spain; b Facultat de Medicina i Ciències de la Salut, Universitat de Barcelona (UB), Carrer Casanova, 143, 08036 Barcelona, Spain; c Centre d'Atenció Primaria Casanova, Consorci d'Atenció Primària de Salut Barcelona Esquerra (CAPSBE), Carrer Rosselló 161, 08036 Barcelona, Spain; d Centre d'Atenció Primaria El Clot, Institut Català de la Salut (ICS), Carrer Concilio de Trento 25, 08018 Barcelona, Spain; e Centre d'Atenció Primaria Numància, Institut Català de la Salut (ICS), Carrer Numància 23, 08029 Barcelona, Spain; f Departament de Malalties Infeccioses, Parc Taulí Hospital Universitari. Institut d'Investigació i Innovació Parc Taulí (I3PT-CERCA), Parc Taulí, 1, 08208 Sabadell-Barcelona, Spain; g Departament de Medicina, Universitat Autònoma de Barcelona (UAB), Avinguda Can Domènech, Edifici M, 08193 Ballaterra-Barcelona, Spain; h Centre d'Atenció Primaria Roger, Institut Català de la Salut (ICS), Carrer Roger, 48-64, 08028 Barcelona, Spain; i Centre d'Atenció Primaria Adrià 5A Marc Aureli, Institut Català de la Salut (ICS), Carrer Vallmajor, 34, 08021 Barcelona, Spain; j Centre d'Atenció Primaria Torelló, Institut Català de la Salut (ICS), Avinguda Pompeu Fabra, 8, 08570 Torelló-Barcelona, Spain; k CIBERINFEC, ISCIII-CIBER de Enfermedades Infecciosas, Instituto de Salud Carlos III, Centro de Investigación Biomédica en Red de Enfermedades Infecciosas, Avenida Monforte de Lemos, 3-5, Pabellón 1 Planta 0, 28029 Madrid, Spain; l Departament de Microbiologia, Hospital Clínic Barcelona, Carrer Villaroel, 170, 08036 Barcelona, Spain.; m Department of Medicine Solna, Karolinska Institutet, Solnavägen 1, 17177 Solna-Stockholm, Sweden; n Department of Infectious Diseases, Karolinska University Hospital, Solnavägen 1, 17177 Solna-Stockholm, Sweden

**Keywords:** Migrants, Prevalence, Screening, Infectious diseases, Imported diseases, Travel-related illnesses

## Abstract

**Background:**

Migrants are disproportionately affected by several infectious diseases differing the risk within migrant groups. This study aimed to estimate the prevalence of six chronic infections in asymptomatic migrants attended at primary care or specialized units where health assessments are offered.

**Methods:**

A multicentric cross-sectional study was conducted to estimate the prevalence of human immunodeficiency virus (HIV), hepatitis B and C virus, *Strongyloides stercoralis, Schistosoma* spp*.*, and *Trypanosoma cruzi* infections in the migrant population who participated in a screening programme implemented at six primary health care centres, and two infectious diseases outpatient clinics in Catalonia, Spain.

**Results:**

Of the 314 recruited migrants, 284 (90.4 %) were tested for at least one infection. The prevalence of the infections was as follows: 1.8 % for HIV, 1.8 % for chronic hepatitis B virus, 14.1 % for previous exposure to a hepatitis B virus infection, 0.4 % for hepatitis C virus infection, 2.6 % for *S.stercoralis* infection, and 7.0 % for *T.cruzi* infection. No cases infected with *Schistosoma* spp*.* were reported. A higher prevalence of HIV, hepatitis B and C virus was observed in Sub-Saharan Africans, whereas a higher prevalence of *S.stercoralis* and *T.cruzi* infections was reported in migrants from Latin-America and the Caribbean.

**Conclusion:**

Findings suggest a high burden of the studied infections, including parasitic infections, in the migrant population with variations within migrant groups based on areas of birth.

## Highlights

• Chronic infections represent a high burden in the migrant population.
• Regional and country-specific prevalence estimations enable a more accurate risk identification.
• Parasitic infections affect migrants from most tropical and sub-tropical areas.

## 1 Introduction

International migration is an increasing and challenging phenomenon. The latest estimates from the International Organization for Migration indicate that in 2020, 3.6 % (281 million) of the global population were migrants (International Organization for Migration 2021). Europe was the largest migrant destination, hosting 87 million international migrants (International Organization for Migration 2021), and Spain is among the top tenth receiving countries with 11.3 % of foreign-born population (International Organization for Migration 2021).

Migrants are disproportionately affected by infectious diseases, differing the risk among migrant groups (Sequeira-Aymar et al., 2021). They present a high burden of undiagnosed human immunodeficiency virus (HIV), hepatitis B virus (HBV), and hepatitis C virus (HCV) infections and tend to have a higher mortality rate when compared to the host population (Schousboe and Wejse, 2021; Lazarus et al., 2019). Some migrant groups present a high prevalence of non-endemic chronic infections such as Chagas disease, strongyloidiasis, and schistosomiasis (Navarro et al., 2022; Asundi et al., 2019). Migrants' vulnerability to infections might be explained by the different levels of exposure and the social determinants of health (SDH) that migrants face in their country of birth, during the migration route, and in the host country (European Centre for Disease Prevention and Control (ECDC) 2018).

Early detection and treatment of these infections can reduce the related morbidity and mortality and control the transmission in the host country (Sequeira-Aymar et al., 2021). In this regard, the feasibility (Gonçalves et al., 2022) and health impact (Sequeira-Aymar et al., 2021) of a multi-infection screening programme targeting migrant populations have been demonstrated in a primary care-based study (Gonçalves et al., 2022).

Nonetheless, most prevalence studies for infectious diseases in migrants are hospital-based studies, which may include symptomatic individuals, and target some vulnerable groups within the migrant population, such as undocumented migrants, asylum seekers, and refugees (Norman et al., 2021; Bocanegra et al., 2014; Vignier et al., 2022; Klok et al., 2021). These approaches hold possible selection bias in the results. Furthermore, systematic reviews and meta-analyses may include studies with varying methodologies, which can introduce other types of biases (Schousboe and Wejse, 2021; Lazarus et al., 2019; Navarro et al., 2022; Asundi et al., 2019; Salvador et al., 2020; Requena-Méndez et al., 2015). Therefore, prevalence studies targeting asymptomatic migrants are fundamental to implement effective screening strategies and immunization programmes by accurately classifying their risk.

This study aimed to estimate the prevalence of six chronic infectious diseases in asymptomatic migrants attended at six primary care centres (PCCs) and two hospitals in Catalonia, Spain and to identify potential associations.

## 2 Material and methods

### 2.1 Study design and participants

This multicentric cross-sectional study was part of the INPREMI project, which implemented a systematic screening programme for multiple infections in migrant populations (Aguilar et al., 2024). The screening programme was carried out at six PCCs and two hospitals in Catalonia, Spain, from January 2018 to July 2020 and included six infections (HIV, HBV, HCV, *Strongyloides stercoralis, Schistosoma* spp., and *Trypanosoma cruzi* infections). These diseases were included because they fulfil the Wilson and Jungner criteria for screening (Wilson and Jungner, 1968) and because all of them had a validated serological test to diagnose the condition. Migrants ≥18 years old who attended the participating centres for any reason were invited to participate. Migrants attended at the hospitals were only recruited if they were referred from non-profit organizations or coming from shelters to perform a health assessment, being therefore asymptomatic individuals or individuals with undifferentiated symptoms. Migrants born in Western Europe, North America, Australia, and New Zealand were excluded from the study (Annex 1) since, for the studied infections, their countries of birth either had a very low prevalence (HIV and viral hepatitis) or were not endemic (*T.cruzi, S.stercoralis* and *Schistosoma* spp. infections).

### 2.2 Study procedures

After signing the corresponding informed consent, a case report form (CRF) with socio-demographic and epidemiological data and blood samples were collected from each participant. HIV, HBV, and HCV serological tests were performed on all recruited individuals, whereas parasitic serological tests were requested according to the individual epidemiological risk of acquisition, determined by the endemicity of the infection at the country of birth, following a systematic review and meta-analysis (Requena-Méndez et al., 2015) and the recommendations from the European Centre for Disease Prevention and Control (ECDC) (European Centre for Disease Prevention and Control (ECDC) 2018). An extra blood sample was collected from each participant for the development of a multiple serological test. These findings have been published elsewhere (Aguilar et al., 2024).

HIV antibodies, the Australian antigen (HBsAg) to identify chronic HBV, the antibody to hepatitis B core antigen (anti-HBc) for exposure to an HBV infection, and HCV total antibodies (anti-HCV) were processed using the Atellica™ IM 1600 System of the Siemens Healthineers commercial house. *S.stercoralis* serology was handled with a commercial Enzyme-Linked Immunosorbent Assay (ELISA) test based on the In Vitro Diagnostic (IVD) *S.stercoralis* crude antigen from *SciMedx®. Schistosoma* spp. serology was processed with a commercial indirect hemagglutination (IHA) test manufactured by *Fumouze®. T.cruzi* serology was performed using the Chemiluminescent Microparticle Immunoassay test (CMIA) from *Architect Abbot®* and it was confirmed with a commercial ELISA with recombinant antigens (BioELISA Chagas, *Biokit®*, Barcelona, Spain). Results for all serology tests were considered positive according to the reference values of Hospital Clínic of Barcelona (Annex 2). Individuals with positive results in any of the serology tests were referred to the corresponding medical specialist.

### 2.3 Data collection and variables

The CRF included: socio-demographic characteristics (country of birth, sex, age, educational level, professional category); living conditions in the country of birth (area-urban/rural, contact with fresh water bodies, contact with animals); factors concerning the migration journey (in transit countries, suffered violence); time of residence in Spain, behavioural factors (sexual practices, drug use); and medical history (comorbidities, immunosuppression, symptoms, and medication) (Annex 3).

Countries of birth were aggregated into areas of birth adapting the international classification of Geo-Sentinel (Field et al., 2010), as follows: Latin-America and the Caribbean (LA), Sub-Saharan Africa (SSA), Northern Africa (NA), and other areas of birth (Annex 1). Age was categorized as young adulthood (18–39 years) and middle-old adulthood (≥40 years). Educational level was defined as lower studies for those with primary studies or illiteracy, secondary studies, and higher studies for individuals with a higher education than secondary school. For professional categories, participants were defined as unemployed (students and unemployed individuals), and employed. The time of residence in Spain was categorized into newly arrived (<5 years) and long-term residing migrants (≥5 years), following the ECDC's definitions (European Centre for Disease Prevention and Control (ECDC) 2018). Finally, a distinction between cannabis use and hard drug use (which included cocaine, heroin, and other recreational drugs) was made.

### 2.4 Data analysis

The primary outcome was the prevalence for each infection defined as the number of diagnoses identified by serological tests among the people who were tested. Data collected on serologies and the results were used to estimate the prevalence for each infection with its respective 95 % confidence interval (95 %CI).

Categorical variables were expressed as relative and absolute frequencies and normally distributed continuous variables were described as means and standard deviations (SD). Pearson's chi-square tests or Fisher's exact tests were performed to evaluate the associations between categorical variables. Associations with the continuous variables were evaluated using the one-way analysis of variance (ANOVA) test. Firth´s regression models were executed to check any possible associations between the outcome variables (e.g., exposure to an HBV infection) and predictor variables (area of birth and other factors). Odds Ratio (OR), adjusted OR (aOR), and 95 %CI were reported. The statistical significance was established at the 5 % level. Stata-IC.16.0 (Stata-16, College-Station, TX, USA) was used as the statistical analysis software for this study.

### 2.5 Ethical considerations

The study was approved by the Ethics Committee of the Hospital Clinic in Barcelona (HCB/2017/0847) and the respective ethic committees of all participating centres. An informed consent was obtained from participants, and the clinical research followed the ethical standards of the Declaration of Helsinki. The study was reported using the Strengthening the Reporting of Observational Studies in Epidemiology (STROBE) guidelines (Annex 4).

## 3 Results

**Fig. 1:**
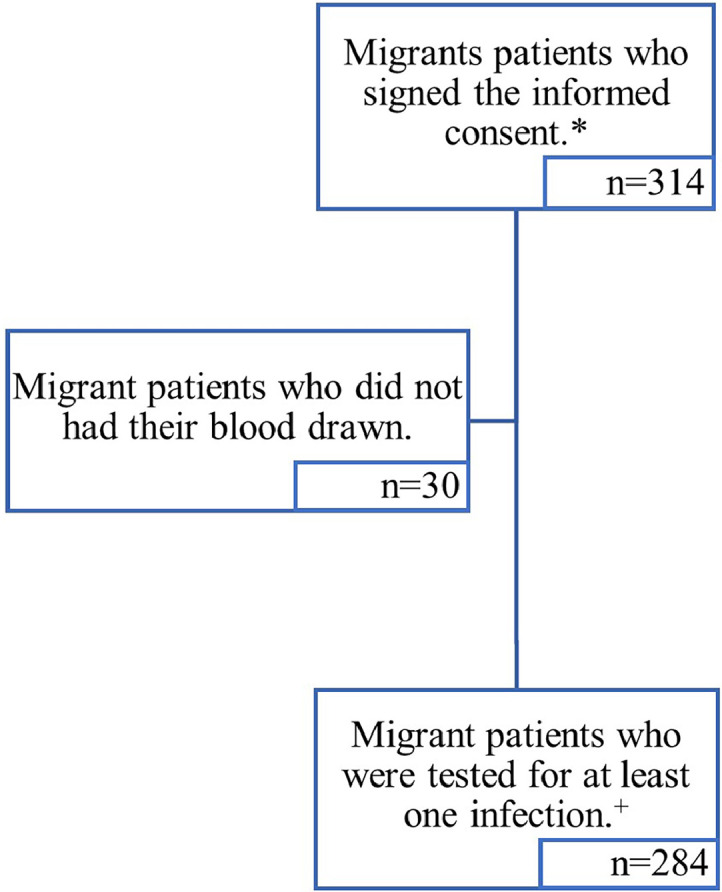
Flow chart of the study population.

A total of 314 participants were included in the study, yet only 284 (90.4 %) were tested for at least one infection (Fig. 1). The highest proportion of migrants were from LA (227/314, 72.3 %), followed by migrants from SSA (44/314, 14.0 %) (Table 1). There were 196/314 (62.4 %) females and the mean age of the study population was 39.4 (SD 0.7), being 168/314 (53.5 %) young adults. Most of the individuals completed secondary studies (153/307, 49.8 %) and were employed (250/309, 80.9 %). Half of the cohort were newly arrived migrants (156/309, 50.5 %). See Table 1 for details of the characteristics of participants by areas of birth.

*Study population; ^+^Population used for the prevalence data estimations

**Table 1:** Characteristics of the sample.

	Total (*N* = 314) n/N ( %)	LA (*N* = 227) n/N ( %)	SSA (*N* = 44) n/N ( %)	NA (*N* = 11) n/N ( %)	Other areas of birth (*N* = 32) n/N ( %)	p-value*
**Total**	314	227 (72.3)	44 (14.0)	11 (3.5)	32 (10.2)	
**Sex** (Female)	196/314 (62.4)	169/227 (74.5)	10/44 (22.7)	3/11 (27.3)	14/32 (43.8)	**<0.001**
**Age** (mean, SD) (*n* = 314)	39.4 (0.7)	39.7 (12.9)	39.7 (10.3)	37.0 (9.1)	38.3 (13.2)	0.169^
**Age categories**						
18–39 years	168/314 (53.5)	123/227 (54.2)	20/44 (45.5)	7/11 (63.6)	18/32 (56.3)	0.628
≥40 years	146/314 (46.5)	104/227 (45.8)	24/44 (54.5)	4/11 (36.4)	14/32 (43.8)	
**Education level**						
Primary studies or illiteracy	48/307 (15.6)	26/225 (11.6)	18/41 (43.9)	3/11 (27.3)	1/30 (3.3)	**<0.001**
Secondary studies	153/307 (49.8)	117/225 (52.0)	20/41 (48.8)	3/11 (27.3)	13/30 (43.3)	
Higher studies	106/307 (34.5)	82/225 (36.4)	3/41 (7.3)	5/11 (45.5)	16/30 (53.3)	
**Professional categories**						
Unemployed	59/309 (19.1)	43/226 (19.0)	6/42 (14.3)	3/11 (27.3)	7/30 (23.3)	0.639
Employed	250/309 (80.9)	183/226 (81.0)	36/42 (85.7)	8/11 (72.7)	23/30 (76.7)	
**Living area in country of birth**						
Urban	180/308 (58.4)	132/226 (58.4)	21/41 (51.2)	7/11 (63.6)	20/30 (66.7)	**0.048**
Rural	57/308 (18.5)	34/226 (15.0)	14/41 (34.2)	2/11 (18.2)	7/30 (23.3)	
Both	71/ 308 (23.1)	60/226 (26.6)	6/41 (14.6)	2/11 (18.2)	3/30 (10.0)	
**Contact with lakes or rivers in country of birth**	178/308 (57.8)	132/226 (58.4)	25/41 (61.0)	5/11 (45.5)	16/30 (53.3)	0.769
**Contact with animals in country of birth**	208/297 (70.0)	160/216 (74.1)	28/41 (68.3)	5/11 (45.5)	15/29 (51.7)	**0.023**
**Transportation**						
Earth	6/309 (1.9)	0/227 (0.0)	2/41 (4.9)	2/11 (18.2)	2/30 (6.7)	**<0.001**
Air	275/309 (89.0)	225/227 (99.1)	21/41 (51.2)	3/11 (27.3)	26/30 (86.7)	
Sea	11/309 (3.6)	0/227 (0.0)	7/41 (17.1)	4/11 (36.4)	0/30 (0.0)	
Earth and Air	6/309 (1.9)	1/227 (0.4)	2/41 (4.9)	1/11 (9.1)	2/30 (6.7)	
Earth and Sea	6/309 (1.9)	0/227 (0.0)	5/41 (12.2)	1/11 (9.1)	0/30 (0.0)	
Air and Sea	2/309 (0.7)	1/227 (0.4)	1/41 (2.4)	0/11 (0.0)	0/30 (0.0)	
Earth, Air and Sea	3/309 (1.0)	0/227 (0.0)	3/41 (7.3)	0/11 (0.0)	0/30 (0.0)	
**In transit countries**						
None	148/304 (14.7)	120/224 (53.6)	13/39 (33.3)	6/11 (54.6)	9/30 (30.0)	**0.004**
1 country	102/304 (33.5)	74/224 (33.0)	11/39 (28.2)	3/11 (27.3)	14/30 (46.7)	
≥2 countries	54/304 (17.8)	30/224 (13.4)	15/39 (38.5)	2/11 (18.2)	7/30 (23.3)	
**Suffered violence**†	39/298 (13.1)	36/218 (16.5)	2/39 (5.1)	0/11 (0.0)	1/30 (3.3)	**0.045**
Sexual violence	5/298 (1.7)	4/218 (1.8)	1/39 (1.6)	-	0/30 (0.0)	
Physical violence	21/298 (7.1)	18/218 (8.3)	2/39 (5.1)	-	1/30 (3.3)	
Human trafficking	1/298 (0.3)	1/218 (0.5)	0/39 (0.0)	-	0/30 (0.0)	
Other types of violence	17/298 (5.7)	17/218 (7.8)	0/39 (0.0)	-	0/30 (0.0)	
Unknown	1/298 (0.3)	1/218 (0.5)	0/39 (0.0)	-	0/30 (0.0)	
**Time residing in Spain in years**						
<5 years	156/309 (50.5)	130/226 (57.5)	8/41 (19.5)	4/11 (36.4)	14/31 (45.2)	**<0.001**
≥5 years	153/309 (49.5)	96/226 (42.5)	33/41 (80.5)	7/11 (63.6)	17/31 (54.8)	
**Unsafe sexual practices**	54/308 (17.5)	37/226 (16.4)	11/41 (26.8)	1/11 (9.1)	5/30 (16.7)	0.406
**Hard drug users**+	7/307 (2.3)	6/224 (2.7)	1/41 (2.4)	0/11 (0.0)	0/31 (0.0)	1.000
**Cannabis users**	15/307 (4.9)	12/224 (5.4)	2/41 (4.9)	1/11 (9.1)	0/31 (0.0)	0.441
**Alcohol consumers**	117/307 (38.1)	100/225 (44.4)	6/40 (15.0)	1/11 (9.1)	10/31 (32.3)	**<0.001**
**Tobacco use**						
Non-smoker	255/307 (83.1)	194/224 (86.6)	26/41 (63.4)	9/11 (81.8)	26/31 (83.9)	**0.013**
Smoker	38/307 (12.4)	21/224 (9.4)	10/41 (24.4)	2/11 (18.2)	5/31 (16.1)	
Ex-smoker	14/307 (4.6)	9/224 (4.0)	5/41 (12.2)	0/11 (0.0)	0/31 (0.0)	
**Comorbidities**†						
Any comorbidity	84/308 (27.3)	67/226 (29.7)	10/41 (24.4)	1/11 (9.1)	6/30 (20.0)	0.381
Diabetes mellitus	13/308 (4.2)	10/226 (4.4)	3/41 (7.3)	0/11 (0.0)	0/30 (0.0)	
Chronic neoplasm	2/308 (0.7)	2/226 (0.9)	0/41 (0.0)	0/11 (0.0)	0/30 (0.0)	
HIV	2/308 (0.7)	1/226 (0.4)	1/41 (2.4)	0/11 (0.0)	0/30 (0.0)	
Chronic lung disease	2/308 (0.7)	2/226 (0.9)	0/41 (0.0)	0/11 (0.0)	0/30 (0.0)	
Inflammatory bowel disease	1/308 (0.3)	1/226 (0.4)	0/41 (0.0)	0/11 (0.0)	0/30 (0.0)	
Chronic liver disease	3/308 (1.0)	3/226 (1.3)	0/41 (0.0)	0/11 (0.0)	0/30 (0.0)	
Chronic nephropathy	3/308 (1.0)	2/226 (0.9)	1/41 (2.4)	0/11 (0.0)	0/30 (0.0)	
Other chronic diseases	68/308 (22.1)	53/226 (23.5)	8/41 (19.5)	1/11 (9.1)	6/30 (20.0)	
Unknown	1/308 (0.3)	1/226 (0.4)	0/41 (0.0)	0/11 (0.0)	0/30 (0.0)	
**Previous TB episode**	7/294 (2.4)	5/218 (2.3)	1/35 (2.9)	0/11 (0.0)	1/30 (3.3)	0.622
**Immunosuppression status**	6/308 (2.0)	5/226 (2.2)	1/41 (2.4)	0/11 (0.0)	0/30 (0.0)	1.000
Transplant	1/308 (0.3)	1/226 (0.4)	0/41 (0.0)	-	-	
Oncological/hematological neoplasm	1/308 (0.3)	1/226 (0.4)	0/41 (0.0)	-	-	
Autoimmune diseases	2/308 (0.7)	2/226 (0.9)	0/41 (0.0)	-	-	
HIV Immunodeficiency	2/308 (0.7)	1/226 (0.4)	1/41 (2.4)	-	-	
**Symptoms**†	89/308 (28.9)	64/226 (28.3)	12/41 (29.3)	4/11 (36.4)	9/30 (30.0)	0.931
Respiratory	17/308 (5.5)	10/226 (4.4)	2/41 (4.8)	2/11 (18.2)	3/30 (10.0)	
Cutaneous	11/308 (3.6)	7/226 (3.1)	3/41 (7.3)	1/11 (9.1)	0/30 (0.0)	
Digestive	49/308 (15.9)	36/226 (15.9)	8/41 (19.5)	0/11 (0.0)	5/30 (15.7)	
Systemic	5/308 (1.6)	3/226 (1.3)	1/41 (2.4)	0/11 (0.0)	1/30 (3.3)	
Neurological	5/308 (1.6)	5/226 (2.2)	0/41 (0.0)	0/11 (0.0)	0/30 (0.0)	
Others	10/308 (3.3)	7/226 (3.1)	2/41 (4.9)	1/11 (9.1)	0/30 (0.0)	
**Current medication**†	103/298 (34.6)	78/218 (35.8)	14/40 (35.0)	3/10 (30.0)	8/30 (26.7)	0.823
Antibiotics	22/298 (7.4)	16/218 (7.3)	4/40 (10.0)	2/10 (20.0)	0/30 (0.0)	
Corticosteroids	8/298 (2.7)	7/218 (3.2)	0/40 (0.0)	0/10 (0.0)	1/30 (3.3)	
Immunosuppressants	1/298 (0.3)	1/218 (0.5)	0/40 (0.0)	0/10 (0.0)	0/30 (0.0)	
Proton-pump inhibitor	8/298 (2.7)	4/218 (1.8)	2/40 (5.0)	2/10 (20.0)	0/30 (0.0)	
Statins	7/298 (2.4)	5/218 (2.3)	1/40 (2.5)	0/10 (0.0)	1/30 (3.3)	
Antihypertensives	32/298 (10.7)	21/218 (9.6)	6/40 (15.0)	1/10 (10.0)	4/30 (13.3)	
Others	54/298 (18.1)	44/218 (20.2)	7/40 (17.5)	1/10 (10.0)	2/30 (6.7)	
Unknown	1/298 (0.3)	0/218 (0.0)	0/40 (0.0)	0/10 (0.0)	1/30 (3.3)	

LA: Latin America and the Caribbean, SSA: Sub-Saharan Africa, NA: Northern Africa, HIV: Human immunodeficiency virus, TB: Tuberculosis;.

Not all questionnaires were fully responded during the interviews explaining why the denominators of the variables vary.

† Categories are not mutually exclusive.

+ Hard drug users include cocaine and other recreational drugs users.

⁎ p-values of the bivariate analysis with Pearson's chi-square test or Fisher's exact test.

^ p-value was estimated with the one-way ANOVA test.

### 3.1 Prevalence

#### 3.1.1 Parasitic infections

The highest prevalence of the parasitic infections was reported for *T.cruzi* (14/192, 7.3 % [95 %CI 3.6–11.0]) (Table 2). Of the 14 *T.cruzi* infected cases, 13 (92.9 %) were migrants from Bolivia and one (7.1 %) from Honduras. There were 7/271 *S.stercoralis* cases resulting in a prevalence of 2.6 % [95 %CI 0.7–4.5]. Six of the *S.stercoralis* infected migrants were from LA (6/198, 3.0 % [95 %CI 0.6–5.4]), and one from SSA (1/38, 2.6 % [95 %CI 0.0–8.0]) (Table 2). Nonetheless, we found no cases infected with *Schistosoma* spp*.* within the 88 tested individuals, 35 migrants were from LA, 38 were from SSA, three were from NA, 10 were from Eastern and Southeast Asia, and two were from Southern Asia and Middle East.

**Table 2:** Prevalence of the infections.

	Total n/N (%, 95 % CI)	LA n/N (%, 95 % CI)	SSA n/N (%, 95 % CI)	NA n/N (%, 95 % CI)	Other areas of birth n/N (%, 95 % CI)	p-value*
***T.cruzi* infection**+	14/192 (7.3, 3.6–11.0)	14/192 (7.3, 3.6–11.0)	–	–	–	–
***S.stercoralis* infection**	7/271 (2.6, 0.7–4.5)	6/198 (3.0, 0.6–5.4)	1/38 (2.6, 0.0–8.0)	0/9	0/26	1.000
				–	–	
**HIV**	5/281 (1.8, 0.2–3.3)	2/204 (1.0, 0.0–2.3)	2/40 (5.0, 0.0–12.1)	0/9	1/28 (3.6, 0.0–10.9)	0.157
				–		
**Chronic HBV** (HBsAg +, HBc +)	5/281 (1.8, 0.2–3.3)	0/205	4/39 (10.3, 0.3–20.2)	0/9	1/28 (3.6, 0.0–10.9)	**0.001**
				–		
		–				
**Exposure to HBV** (HBsAg ±, HBc +)	40/284 (14.1, 10.0–18.2)	4/207 (1.9, 0.0–3.8)	30/41 (73.2, 59.0–87.3)	0/8	6/28 (21.4, 5.2–37.6)	**<0.001**
				–		
**HCV**	1/284 (0.4, 0.0–1.0)	0/207	1/41 (2.4, 0.0–7.4)	0/9	0/27	0.271
		–		–	–	

LA: Latin America and the Caribbean, SSA: Sub-Saharan Africa, NA: Northern Africa, HIV: Human immunodeficiency virus, HBV: Hepatitis B virus, HBsAg: Australian antigen, HCV: Hepatitis C virus;.

Schistosomiasis was excluded due to the absence of identified cases.

+ Only people from endemic areas of Latin-America were tested for *T.cruzi*.

⁎ p-values of the bivariate analysis with Pearson's chi-square test or Fisher's exact test.

#### 3.1.2 HIV and viral hepatitis

Five of the 281 migrants tested for HIV were positive presenting a prevalence of 1.8 % [95 %CI 0.2–3.3], two were born in LA (2/204, 1.0 % [95 %CI 0.0–2.3]), two in SSA (2/40, 5.0 % [95 %CI 0.0–12.1]), and one in Southern Asia and the Middle East (Table 2). For viral hepatitis, 1/284 (0.4 % [95 %CI 0.0–1.0]) migrants were infected with HCV, 5/28 (1.8 % [95 %CI 0.2–3.3]) presented a chronic HBV infection, and 40/284 (14.1 % [95 %CI 10.0–18.2]) were previously exposed to HBV. Among the HBsAg positive cases, four were migrants from SSA (4/39, 10.3 % [95 %CI 0.3–20.2]), and the other case was a migrant from other areas of birth (1/28, 3.6 % [95 %CI 0.0–10.9]), specifically from Southeast Asia (p-value=0.001). SSA migrants (30/41, 73.2 % [95 %CI 59.0–87.3]) reported the highest prevalence of exposure to HBV, followed by migrants from other areas of birth (6/28, 21.4 % [95 %CI 5.2–37.6]) (Table 2).

### 3.2 Factors associated with chronic infections

#### 3.2.1 Parasitic infections

A higher proportion of *T.cruzi* infections were observed in males (7/52, 13.5 %; *p* = 0.050), and migrants with primary studies or illiteracy (3/20, 15.0 %; *p* = 0.028) (Table 3). Most of the diagnosed individuals lived in rural areas (4/29, 13.8 %; *p* = 0.013) in their country of birth and were considered long-term residing migrants (10/87, 11.5 %; *p* = 0.041). Moreover, the majority of the migrants infected with *S.stercoralis* had primary studies or illiteracy (4/39, 10.3 %; *p* = 0.008), and lived in both urban and rural areas in their country of birth (4/64, 6.3 %; *p* = 0.029) (Table 3). All cases were reported in long-term residing migrants (7/137, 5.1 %; *p* = 0.009).

**Table 3:** Associations between the social determinants of health and the infectious diseases.

	***T.cruzi* infection**^ **(*N*=192)**		***S.stercoralis* infection (*N*=271)**		**HIV (*N*=281)**		**Chronic HBV (*N*=281)**	
	n/N (%)	p-value*	n/N ( %)	p-value*	n/N ( %)	p-value*	n/N ( %)	p-value*
**Area of birth**								
LA	14/192 (7.3)	–	6/198 (3.0)	1.000	2/204 (1.0)	0.157	0/205 (0.0)	**0.001**
SSA	–		1/38 (2.6)		2/40 (5.0)		4/39 (10.3)	
NA	–		0/9 (0.0)		0/9 (0.0)		0/9 (0.0)	
Other areas of birth	–		0/26 (0.0)		1/28 (3.6)		1/28 (3.6)	
**Sex**								
Male	7/52 (13.5)	**0.050**	4/106 (3.8)	0.271	4/108 (3.7)	0.074	5/109 (4.6)	**0.008**
Female	7/140 (5.0)		3/165 (1.8)		1/173 (0.6)		0/172 (0.0)	
**Age categories**								
18–39 years	6/100 (6.0)	0.473	1/139 (0.7)	0.061	2/145 (1.4)	0.676	1/145 (0.7)	0.201
≥40 years	8/92 (8.7)		6/132 (4.6)		3/136 (2.2)		4/136 (2.4)	
**Education level**^1^								
Primary studies or illiteracy	3/20 (15.0)	**0.028**	4/39 (10.3)	**0.008**	0/40 (0.0)	0.812	3/39 (7.7)	**0.004**
Secondary studies	10/103 (9.7)		3/138 (2.2)		3/138 (2.2)		0/139 (0.0)	
Higher studies	1/67 (1.5)		0/88 (0.0)		1/96 (1.0)		1/96 (1.0)	
**Professional categories**^2^								
Unemployed	2/35 (5.7)	1.000	0/49 (0.0)	0.355	1/52 (1.9)	0.568	0/50 (0.0)	0.589
Employed	12/156 (7.7)		7/217 (3.2)		3/224 (1.3)		5/226 (2.2)	
**Living area in country of birth**^3^								
Urban	3/109 (2.8)	**0.013**	1/155 (0.7)	**0.029**	3/161 (1.9)	1.000	2/163 (1.2)	0.365
Rural	4/29 (13.8)		2/47 (4.3)		0/47 (0.0)		0/47 (0.0)	
Both	7/53 (13.2)		4/64 (6.3)		1/67 (1.5)		2/65 (3.1)	
**Contact with rivers or lakes**^4^								
No	5/82 (6.1)	0.392	2/116 (1.7)	0.342	–	–	–	–
Yes	9/109 (8.3)		5/150 (3.3)					
**In transit countries**^5^ None								
1 country	9/99 (9.1)	0.361	4/127 (3.2)	1.000	2/131 (1.5)	0.119	1/131 (0.8)	0.503
≥2 countries	4/67 (6.0)		2/94 (2.1)		0/94 (0.0)		2/95 (2.1)	
	0/23 (0.0)		1/41 (2.4)		2/46 (4.4)		1/45 (2.2)	
**Suffered violence**^6^								
No	11/151 (7.3)	0.113	5/221 (2.3)	0.590	3/228 (1.3)	0.454	4/229 (1.8)	0.547
Yes	0/32 (0.0)		1/35 (2.9)		1/37 (2.7)		0/37 (0.0)	
**Time residing in Spain in years**^7^								
<5 years	4/104 (3.9)	**0.041**	0/130 (0.0)	**0.009**	1/131 (0.8)	0.624	0/134 (0.0)	0.069
≥5 years	10/87 (11.5)		7/137 (5.1)		3/145 (2.1)		4/142 (2.8)	
**Unsafe sexual practices**^8^								
No	13/155 (8.4)	0.217	6/218 (2.8)	0.631	3/226 (1.3)	0.546	4/226 (1.8)	0.454
Yes	1/36 (2.8)		1/48 (2.1)		1/49 (2.0)		0/49 (0.0)	
**Hard drug users**+^,^^9^								
No	12/185 (6.5)	1.000	7/260 (2.7)	1.000	4/267 (1.5)	1.000	4/267 (1.5)	1.000
Yes	0/4 (0.0)		0/5 (0.0)		0/7 (0.0)		0/7 (0.0)	
**Cannabis users**^10^								
No	12/180 (6.7)	1.000	7/254 (2.8)	1.000	3/260 (1.2)	0.190	4/260 (1.5)	1.000
Yes	0/9 (0.0)		0/11 (0.0)		1/14 (7.1)		0/14 (0.0)	
**Comorbidities**^11^								
No	11/137 (8.0)	0.425	5/198 (2.5)	0.571	2/201 (1.0)	0.294	3/200 (1.5)	0.700
Yes	3/54 (5.6)		2/68 (2.9)		2/74 (2.7)		1/75 (1.4)	
**Previous TB episode**^12^								
No	12/182 (6.6)	0.764	6/250 (2.4)	0.887	3/256 (1.2)	0.922	4/256 (1.6)	0.897
Yes	0/4 (0.0)		0/5 (0.0)		0/7 (0.0)		0/7 (0.0)	
**Immunosuppression status**^13^								
No	14/189 (7.4)	0.858	7/264 (2.7)	0.948	2/270 (0.7)	**0.002**	4/271 (1.5)	0.943
Yes	0/2 (0.0)		0/2 (0.0)		2/5 (40.0)		0/4 (0.0)	
**Symptoms**^14^								
No	11/136 (8.1)	0.386	5/189 (2.7)	0.672	2/195 (1.0)	0.332	3/197 (1.5)	0.681
Yes	3/55 (5.5)		2/77 (2.6)		2/80 (2.5)		1/78 (1.3)	
**Current medication**^15^								
No	8/115 (7.0)	0.520	3/166 (1.8)	0.355	3/169 (1.8)	0.541	3/170 (1.8)	0.548
Yes	4/68 (5.9)		3/90 (3.3)		1/96 (1.0)		1/95 (1.1)	

HIV: Human immunodeficiency virus, HBV: Hepatitis B virus, HCV: Hepatitis C virus, TB: Tuberculosis;.

Not all questionnaires were fully responded during the interviews explaining why the denominators of the variables vary.

+ Hard drug users include cocaine and other recreational drugs users.

⁎ p-values of the bivariate analysis with Pearson's chi-square test or Fisher's exact test.

^ Only people from endemic areas of Latin-America were tested for *T.cruzi*;.

1 7 missing values.

2 5 missing values.

3 6 missing values.

4 6 missing values.

5 10 missing values.

6 16 missing values.

7 5 missing values.

8 6 missing values.

9 7 missing values.

10 7 missing values.

11 6 missing values.

12 20 missing values.

13 6 missing values.

14 6 missing values.

15 16 missing values.

#### 3.2.2 HIV and viral hepatitis

There were 2/5 (40.0 %, *p* = 0.002) immunosuppressed migrants among those infected with HIV. The only HCV infection diagnosis was observed in a 45-year-old female born in SSA. All five chronic HBV cases were reported in males (5/109, 4.6 %; *p* = 0.008). Three of the four chronic HBV cases that reported their education level had primary studies or illiteracy (3/39, 7.7 %; *p* = 0.004) (Table 3). Besides, a higher proportion of males (31/109, 28.4 %; *p* < 0.001) and migrants in their middle-old adulthood (27/137, 19.7 %; *p* = 0.009) had been previously exposed to an HBV infection (Table 4). Primary studies or illiteracy (18/40, 45.0 %; *p* < 0.001) and residing in Spain ≥5 years (33/144, 22.9 %; *p* < 0.001) were associated with exposure to HBV (Table 4).

**Table 4:** Associations between the social determinants of health and exposure to an HBV infection.

	Exposure to an HBV infection (*N* = 284)				
			Unadjusted model	Adjusted model	
	n/N (%)	p-value*	OR (95 % CI)	aOR (95 % CI)	p-value^
**Area of birth**					
LA	4/207 (1.9)	**<0.001**	reference	reference	**<0.001**
SSA	30/41 (73.2)		119.9 (37.8–380.4)	87.9 (19.3–401.1)	
NA	0/8 (0.0)		2.7 (0.1–53.5)	0.6 (0.0–16.9)	
Other areas of birth	6/28 (21.4)		13.1 (3.6–46.9)	13.0 (2.9–58.8)	
**Sex**					
Male	31/109 (28.4)	**<0.001**	reference	reference	**0.058**
Female	9/175 (5.1)		0.1 (0.1–0.3)	0.3 (0.1–1.0)	
**Age categories**					
18–39 years	13/147 (8.8)	**0.009**	reference	reference	**0.032**
≥40 years	27/137 (19.7)		2.5 (1.2–5.0)	3.9 (1.1–13.6)	
**Education level**^1^				0.3	
Primary studies or illiteracy	18/40 (45.0)	**<0.001**	reference	reference	**0.012**
Secondary studies	13/140 (9.3)		0.1 (0.1–0.3)	0.1 (0.0–0.5)	
Higher studies	6/97 (6.2)		0.1 (0.0–0.2)	0.4 (0.1–1.7)	
**Professional categories**^2^					
Unemployed	5/52 (9.6)	0.501	reference	–	–
Employed	33/227 (14.5)		1.5 (0.6–3.9)		
**In transit countries**^3^					
None	14/132 (10.6)	0.274	reference	–	–
1 country	12/96 (12.5)		1.2 (0.5–2.7)		
≥2 countries	9/46 (19.6)		2.1 (0.8–5.1)		
**Suffered violence**^4^					
No	32/231 (13.9)	0.190	reference	–	–
Yes	2/37 (5.4)		0.4 (0.1–1.6)		
**Time residing in Spain in years**^5^					
<5 years	4/135 (3.0)	**<0.001**	reference	reference	**0.034**
≥5 years	33/144 (22.9)		8.8 (3.2–24.2)	4.2 (1.1–16.0)	
**Unsafe sexual practices**^6^					
No	28/228 (12.3)	0.356	reference	–	–
Yes	9/50 (18.0)		1.6 (0.7–3.6)		
**Hard drug users**+**^,^**^7^					
No	35/270 (13.0)	0.627	reference	–	–
Yes	1/7 (14.3)		1.5 (0.3–9.4)		
**Cannabis users**^8^					
No	35/263 (13.3)	1.000	reference		
Yes	1/14 (7.1)		0.7 (0.1–4.0)		
**Comorbidities**^9^					
No	26/202 (12.9)	0.697	reference	–	–
Yes	11/76 (14.5)		1.2 (0.6–2.5)		
**Immunosuppression status**^10^					
No	36/273 (13.2)	0.513	reference	–	–
Yes	1/5 (20.0)		2.2 (0.3–14.2)		

HBV: Hepatitis B virus, LA: Latin America and the Caribbean, SSA: Sub-Saharan Africa, NA: Northern Africa, OR: odds ratio, aOR: adjusted odds ratio;.

Not all questionnaires were fully responded during the interviews explaining why the denominators of the variables vary.

Variables not considered in the final model.

The multivariate model was adjusted by area of birth, sex, age, education level and time residing in Spain in years.

+ Hard drug users include cocaine and other recreational drugs users.

⁎ p-value of the bivariate analysis with Pearson's chi-square test or Fisher's exact test.

^ p-value of the adjusted model;

1 7 missing values.

2 5 missing values.

3 10 missing values.

4 16 missing values.

5 5 missing values.

6 6 missing values.

7 7 missing values.

8 7 missing values.

9 6 missing values.

10 6 missing values.

In the multivariable model, migrants from SSA (aOR: 87.9 [95 %CI 19.3–401.1]) and other areas of birth (aOR: 13.0 [95 %CI 2.9–58.8]) had higher odds of being exposed to HBV than migrants from LA after adjusting by sex, age, educational level, and time of residence in Spain (*p* < 0.001) (Table 4). When adjusting the model, females (aOR: 0.3 [95 %CI 0.1–1.0]; *p* = 0.058) and migrants with secondary studies (aOR: 0.1 [95 %CI 0.0–0.5]; *p* = 0.012) had lower odds of previous exposure to HBV than males and participants with lower educational studies. Migrants in their middle-old adulthood (aOR: 3.9 [95 %CI 1.1–13.6]; *p* = 0.032) and long-term residing migrants (aOR: 4.2 [95 %CI 1.1–16.0]; *p* = 0.034) presented higher odds of previous exposure to HBV than young adults and newly arrived migrants, respectively, in the adjusted model (Table 4).

Due to the low number of observations, regression models were not explored for other infections.

## 4 Discussion

Overall, we found a high prevalence of chronic infections in the migrant population, especially in *T.cruzi* and previous exposure to an HBV infection. Migrants born in countries of SSA and LA were more vulnerable to infectious diseases. However, these results should be interpreted with caution since the sample was mostly represented by migrants born in LA (∼75 %), following the trend of the migrant population residing in Barcelona (Spain) (Statistical Institute of Catalonia 2022), where most of the participating centres were located.

Interestingly, we were unable to detect any cases infected with *Schistosoma* spp. despite that more than half of the participants had contact with freshwater bodies in their country of birth. This fact might be related to the low sample size of migrants born in SSA and Asian endemic countries (European Centre for Disease Prevention and Control (ECDC) 2018). The prevalence of *S.stercoralis* infection in our study was in line with recent experts’ calculations for the World Health Organization (WHO) European Region (2.8 %) (Buonfrate et al., 2020). However, a meta-analysis of migrants living in Spain indicated a 14 % prevalence where the majority of the included studies were conducted at Tropical Medicine Units (Salvador et al., 2020). Differences in prevalence estimates were observed in comparison to prior studies due to differences in sample composition, having recruited a higher number of Asian and African migrants (Asundi et al., 2019; Salvador et al., 2020). Therefore, future studies should consider the inclusion of migrants from origins that were under represented in our sample to estimate the burden of *S.stercoralis* better among these populations.

We found a prevalence of *T.cruzi* infection similar to estimates observed in non-hospital settings (8.7 %) in a recent systematic review and meta-analysis (Requena-Méndez et al., 2015). We also found that Bolivians had the highest *T.cruzi* infection prevalence, followed by people from Honduras, as previous studies had demonstrated (Navarro et al., 2022; Requena-Méndez et al., 2015). Therefore, testing interventions at strategic points, such as primary care and community level, should focus on migrants born in these high-prevalent countries to control the Chagas disease epidemic in non-endemic areas.

Furthermore, low educational levels and living conditions (living in rural or both rural and urban areas) in the country of birth were associated with both *T.cruzi* and *S.stercoralis* infections. Similar results have been observed in studies conducted in endemic areas (Fernandez et al., 2019; Beknazarova et al., 2016) and Spain (Ramos-Sesma et al., 2021; Ramos-Sesma et al., 2020), suggesting that both neglected tropical diseases share socio-economical and geographical risk factors (Salvador et al., 2020; Requena-Méndez et al., 2015). Both infections were more prevalent in long-term residing migrants (≥5 years), implying that infected individuals can be asymptomatic and carry the infection for a long time. Since both infections can develop severe complications under certain circumstances, such as immunosuppression, our findings support the idea that screening strategies in non-endemic settings should target all individuals coming from endemic countries (Navarro et al., 2022).

The HIV prevalence was similar to previous estimates made at primary care in Barcelona, Spain (1.2 %) (Hladun et al., 2014). Moreover, a systematic review of HIV prevalence in migrants concluded that migrants living in Europe and coming from SSA had a high burden of HIV infection (>3.0 %), while a lower prevalence was estimated for LA migrants (0.7 %) (Schousboe and Wejse, 2021), which is also similar to the data reported in our study. The heterogeneity within the migrant population should be considered when planning health policies on HIV control strategies for better use of limited resources. These strategies should target migrants at higher risk of infection instead of including individuals coming from low-endemic areas, as most LA countries are considered (Sequeira-Aymar et al., 2021; Schousboe and Wejse, 2021).

Migration itself is not a risk factor for the acquisition of HIV, however, factors related to migration can make an individual vulnerable to the infection (Schousboe and Wejse, 2021). We did not find any association between HIV and factors (violence during migration journey, unsafe sexual practices, drug use) that have been previously associated with the infection (Klok et al., 2021; Prestileo et al., 2022), although the small sample size may have precluded properly assessing this association. Further, 75.0 % of the HIV cases that reported time of residence in Spain were identified as long-time residing migrants, supporting the idea of an increased risk of HIV acquisition after migrating to European countries (European Centre for Disease Prevention and Control, WHO Regional Office for Europe 2021). Expectedly, most HIV cases in our study were diagnosed in males, as these are often associated with a higher burden of infection [(European Centre for Disease Prevention and Control (ECDC) 2018; Klok et al., 2021)]. Hence, all these factors might be considered to identify migrants at higher risk and to conduct educational and screening interventions focusing on these vulnerable groups.

The HCV prevalence found in our study was lower than in previous studies. These findings must be taken with caution, however, they might suggest that current control strategies to decrease the viral hepatitis burden are working (Lazarus et al., 2019). A considerable difference in HCV prevalence was observed within our results and previous studies, such as a meta-analysis (1.6 %) (Lazarus et al., 2019), hospital-based studies (2.6–4.2 %) (Norman et al., 2021; Bocanegra et al., 2014), and non-hospital settings (1.8–3.3 %) (Hladun et al., 2014; Alarcón-Linares et al., 2019), where the study population had a higher representation of migrants from high-prevalent countries. The highest HCV prevalence has been mostly described in migrants from Europe (mostly Eastern Europeans and Italians) and SSAs, followed by NAs, while migrants coming from LA tend to present a lower prevalence (Lazarus et al., 2019; Bocanegra et al., 2014; Hladun et al., 2014; Alarcón-Linares et al., 2019). The only case in our study was observed in a migrant born in SSA, yet none of the migrants coming from LA were diagnosed. In line with the WHO's global hepatitis strategy to achieve the target of HCV elimination by 2030 (World Health Organization 2022), although our results cannot be conclusive, they can recommend the promotion of HCV screening, particularly in migrants coming from SSA countries.

Further, the chronic HBV prevalence data of our study is comparable with two cross-sectional studies conducted in Spain, 1.4 % in a population-based study (Cuadrado et al., 2020) and 2.6 % in a primary care-based study (Hladun et al., 2014); and with two other studies targeting undocumented migrants, one in France (3.1 %) (Vignier et al., 2022) and the other in the Netherlands (2.5 %) (Klok et al., 2021). Other studies suggested a much higher prevalence, such as a European systematic review (Cuadrado et al., 2020) and two Spanish hospital-based studies (Norman et al., 2021; Bocanegra et al., 2014), which reported an HBsAg prevalence of around 6 %. On the other hand, prevalence rates rise up to 18.0 % when considering exposure to HBV estimated in Spanish non-hospital settings (Hladun et al., 2014; Cuadrado et al., 2020), congruent to our data. Our findings suggest that migrants coming from SSA are significantly affected by chronic HBV and have been previously exposed to the infection, presenting higher odds when compared to other geographical areas. As this tendency has also been observed in different low-endemic countries in Europe, experts agree that HBV screening strategies should target migrant populations, particularly those born in SSA countries (Sequeira-Aymar et al., 2021; Vignier et al., 2022; Klok et al., 2021; Hladun et al., 2014; Prestileo et al., 2022).

In Spain, the incidence of HBV has decreased in the last decades due to the expanded vaccination programmes implemented in 1992 (Arístegui Fernández et al., 2015). Nonetheless, migrants residing in Spain can come from countries where vaccination strategies are not yet largely implemented. They can also be exposed to different risk factors during the migration journey, making them more vulnerable to the infection than the autochthonous population (Stroffolini et al., 2021). This can increase the host country's HBV prevalence and become a public health challenge. Therefore, identifying individuals at risk of infection is essential to prevent the transmission of the infection and control disease progression by vaccinating the nonimmunized and treating those already infected, as well as promoting migrants’ health. In accordance with previous studies (Klok et al., 2021; Cuadrado et al., 2020), we found that males with lower educational levels were associated with presenting both exposure to HBV and chronic HBV infection. Also, we found an association between long residing migrants and exposure to HBV, and therefore, immunization as it has been previously reported in undocumented migrants residing in the Netherlands (Klok et al., 2021). Finally, as Cuadrado and colleagues described (Cuadrado et al., 2020), older migrants were more commonly exposed to HBV than younger adults due to the highest possibility of being vaccinated against the infection in the latter group.

Integrated screening of infectious diseases targeting migrant patients based on an individual assessment has already been proven effective (Sequeira-Aymar et al., 2021). Nonetheless, migrant populations are considered a homogenous group in most of the screening guidelines. Policymakers in the public health sector should consider individualizing the screening decision process and, therefore, take into account the prevalence estimations of infections in migrants to identify and treat individuals at risk at an early stage, optimizing scarce resources and decreasing health-related costs. The area of birth and other factors associated with a higher risk of infection may be good indicators to make a screening decision. For example, the parasitic infection burden is usually attributed to African migrants, however, these infections disproportionally affect migrants coming from other areas in LA and Asia. Conversely, HIV and viral hepatitis infections are more globally distributed. Thus, migrants born in intermediate and high-prevalent countries (European Centre for Disease Prevention and Control (ECDC) 2018) are susceptible to these infections. Also, information on the levels of anti-HBc could support the promotion of vaccination strategies targeting migrant groups with a higher risk of acquiring the infection and, at the same time, exclude individuals already exposed to the infection.

### 4.1 Limitations

The primary care setting offers a more representative sample of the migrant population than hospital-based studies. In our study, individuals were also recruited in two hospitals (18 %), and although researchers were instructed to recruit only asymptomatic migrants or migrants with nonspecific symptoms, these settings could have introduced potential referral bias. In any case, the purpose of this screening programme was to increase the detection of infectious diseases, irrespective of the symptoms presented by the migrant individuals.

Besides, the sample and the selection of the centres were not randomized, thus the estimated prevalence is only representative of the migrant population attended at the participating centres.

Due to the composition of the sample, under- or over-estimations of the prevalence of the different infections could have been reported. In addition, the small sample size of the study limited the seroprevalence comparison among groups due to the low number of infectious disease cases reported in migrants from different areas of birth. The sample size affected the precision of the seroprevalence estimations with wide 95 %CI intervals for some infections, limiting its possible comparison with other populations, such as the autochthonous population. The low number of events for most infections limited the multivariate analysis, with only one possible model, exposure to HBV. Furthermore, the estimates of this model could be biased due to the few events and small sample size, which has limited the power to detect differences if they exist.

Regarding the number of tests performed, only one HBsAg test was done instead of the two recommended, within at least a six-month difference, to confirm the chronicity of an infected individual. Similarly, polymerase chain reaction tests to confirm HCV chronic infections were not performed. On the other side, even though tuberculosis is prevalent in the migrant population, this infection was not included in the prevalence study due to the limitation of adding it to a multiplex serological test (Aguilar et al., 2024), which was one of the objectives of our research work in order to streamline the screening.

Other limitations were the loss of follow-up of some participants during the Covid-19 pandemic and the possible underreporting of certain behavioural risk factors as a result of the face-to-face interviews.

## 5 Conclusions

A high prevalence of the studied chronic infectious diseases was observed in the migrant population. The country of birth, in particular, and other factors migrants were exposed to in their country of birth, during their migration journey, and in their country of residence were factors associated with the infections. Therefore, the heterogeneity of the migrant population must be considered when planning and implementing preventive strategies.

## Funding

This work was supported by the grant “Fondo de Investigación para la Salud (FIS)” [PI17/02,020] from the Instituto de Salud Carlos III (ISCIII) co-financed by the European Regional Development Fund (FEDER) from the European Union. ISGlobal acknowledges support from the grant [CEX2018–000,806-S] funded by MCIN/AEI/10.13039/501,100,011,033, and support from the 10.13039/501100002809Generalitat de Catalunya through the CERCA Programme. ARM is funded by the Strategic Research Programme in Epidemiology at Karolinska Institutet. ACz is funded by ISCIII co-financed by FEDER from the European Union through the “Contratos predoctorales de formación en investigación (PFIS)” [FI22/00,156].

The funders of the study had no role in the study design, data collection, data analysis, data interpretation, or writing of the manuscript.

## Data availability

Data may be available from the corresponding author upon reasonable request.

## CRediT authorship contribution statement

**Angeline Cruz:** Writing – review & editing, Writing – original draft, Project administration, Formal analysis. **Angela Martínez-Perez:** Writing – review & editing, Writing – original draft, Methodology, Investigation, Conceptualization. **Alex Almuedo-Riera:** Writing – review & editing, Writing – original draft, Project administration, Methodology, Investigation, Conceptualization. **Carme Roca Saumell:** Writing – review & editing, Investigation. **Marina Gigante Lopez:** Writing – review & editing, Investigation. **Oriol Gasch:** Writing – review & editing, Investigation, Funding acquisition, Conceptualization. **Gemma Falcó:** Writing – review & editing, Investigation. **Ana Jiménez-Lozano:** Writing – review & editing, Investigation. **Consol Sanchez-Collado:** Writing – review & editing, Investigation. **Julio Alonso-Padilla:** Writing – review & editing, Methodology, Conceptualization. **Juan Carlos Hurtado:** Writing – review & editing, Investigation. **Miriam J Álvarez-Martínez:** Writing – review & editing, Investigation. **Aina Casellas:** Writing – review & editing, Formal analysis, Data curation. **Ana Requena-Méndez:** Writing – review & editing, Writing – original draft, Methodology, Funding acquisition, Formal analysis.

## Declaration of competing interest

The authors declare that they have no known competing financial interests or personal relationships that could have appeared to influence the work reported in this paper.

## Appendix Supplementary materials

Image, application 1
