# Supplementary material for: Estimating the prevalence of chronic infections among asymptomatic migrants: results of a screening programme in Catalonia, Spain
Source: J Migr Health. 2024 Oct 30;10:100278. doi: 10.1016/j.jmh.2024.100278 (PMC11585815; doi:10.1016/j.jmh.2024.100278)
Supplement: Supplementary file 1 [file mmc1.docx]

**Supplementary files**

**Annex 1.** Classification of areas of birth

| **Areas of birth*** | **Sub-areas of birth^** | **Countries** |
| --- | --- | --- |
| Latin-America and the Caribbean (LA) | Caribbean, Central and South America | Anguilla, Antigua and Barbuda, Netherlands Antilles, Argentina, Aruba, The Bahamas, Barbados, Belize, Bermuda Islands, Bolivia, Bosnia and Herzegovina, Brazil, Cayman Islands, Chile, Colombia, Costa Rica, Cuba, Dominica, Dominican Republic, Ecuador, El Salvador, Falkland Islands, French Guiana, Grenada, Guadeloupe, Guatemala, Guyana, Haiti, Honduras, Jamaica, Martinique, Mexico, Montserrat, Nicaragua, Panama, Paraguay, Peru, Puerto Rico, Saint Christopher and Nevis, Saint Lucia, Saint Vincent and the Grenadines, South Georgia and the South Sandwich Islands, Surinam, Trinidad and Tobago, Turks and Caicos Islands, Uruguay, Venezuela, Virgin Islands (USA), and Virgin Islands (Great Britain) |
| Northern Africa (NA) | Northern Africa | Algeria, Egypt, Libya, Morocco, Tunisia, and Western Sahara |
| Sub-Saharan Africa (SSA) | Sub-Saharan Africa | Angola, Botswana, Benin, Burkina Faso, Burundi, Cameroon, Cape Verde, Central African Republic, Chad, Comoros, Congo, Democratic Republic of Congo, Djibouti, Equatorial Guinea, Eritrea, Ethiopia, Gabon, Gambia, Ghana, Guinea, Guinea Bissau, Ivory Coast, Kenya, Lesotho, Liberia, Madagascar, Malawi, Mali, Mauritia, Mauritania, Mayotte, Mozambique, Namibia, Niger, Nigeria, Republic of South Africa, Réunion, Rwanda, Saint Helena, Saint Thomas and Prince, Senegal, Seychelles, Sierra Leone, Somalia, Sudan, Swaziland, United Republic of French Tanzania, Togo, Uganda, Zambia, and Zimbabwe |
| Other areas of birth | Eastern Europe | Armenia, Azerbaijan, Belarus, Czech Republic, Estonia, Russian Federation, Hungary, Kazakhstan, Kyrgyzstan, Latvia, Lithuania, Moldova, Poland, Romania, Serbia, Slovakia, Tajikistan, Ukraine People´s Republic, and Uzbekistan |
|  | Southern Asia and Middle East | Afghanistan, Saudi Arabia, Bahrain, India, Iraq, Islamic Republic of Iran, Israel, Jordan, Kuwait, Lebanon, Oman, Pakistan, Qatar, Syria, Territory Occupied by Palestine, Turkmenistan, United Arab Emirates, and Yemen |
|  | Eastern and Southeast Asia | Bangladesh, Bhutan, Brunei, Cambodia, China, Republic of Korea, Democratic People’s Republic of Korea, Hong Kong, Indonesia, Japan, Philippines, Lao People’s Democratic Republic, Macau, Malaysia, Mongolia, Myanmar, Nepal, Singapore, Sri Lanka, Thailand, Taiwan, East Timor, and Vietnam |
| Westernized countries**^+^** | Northern Europe | Aland Islands, Austria, Belgium, Bouvet Island (Norway), Denmark, Faroe Islands, Finland, Georgia, Germany, Guernsey, Iceland, Ireland, Isle of Man, Jersey, Liechtenstein, Luxembourg, Monaco, Norway, Netherlands, United Kingdom, Sweden, Switzerland, Svalbard and Jan Mayen, and French Southern and Antarctic Land |
|  | Southern Europe | Albania, Andorra, Bulgaria, Croatia, Cyprus, Slovenia, France, Gibraltar, Greece, Italy, North Macedonia, Malta, Montenegro, Portugal, San Marino, Turkey, and Vatican City |
|  | Anglo-Saxon America | Canada,nevi United States of America, United States Minor Outlying Islands, and Saint-Pierre and Miquelon |
|  | Oceania | Australia, British Indian Ocean Territory, Christmas Island, Cocos Islands, Cook Islands, Federated States of Micronesia, Fiji, French Polynesia, Guam, Heard and McDonald Islands, Kiribati, Maldives, Northern Mariana Islands, Marshall Islands, Nauru, Niue, Norfolk Island, New Caledonia, New Zealand, Palau, Papa New Guinea, Pitcairn, Solomon, Samoa, American Samoa, Tokelau, Tonga, Tuvalu, Vanuatu, and Wallis and Futuna |
| *Classification used for the analysis; +Area not included in the analysis; ^Inspired by the GeoSentinel classification but has been adapted by The Foundation University Institute for Primary Health Care Research Jordi Gol i Gurina (IDIAPJGol) | | |

**Annex 2.** Reference values for positive serologies of the Hospital Clínic of Barcelona

| **Infections** | **Antibodies/Antigens** | **Tests** | **Commercial house** | **Serologies cut-offs** |
| --- | --- | --- | --- | --- |
| **HIV** | VIH1/2 antibodies and antigen p24 | CLIA test | Atellica® IM HIV Ag/Ab Combo of the Siemens Healthineers commercial house | ≥ 1.0 |
| **Hepatitis B virus** | anti-HBc | CLIA test | Atellica IM Anti‑Hepatitis B core Total (HBcT) of the Siemens Healthineers commercial house | ≥ 0.5 |
|  | HBsAg | CLIA test | Atellica IM Hepatitis B surface  Antigen II (HBsII) of the Siemens Healthineers commercial house | ≥ 1.0 |
| **Hepatitis C virus** | anti-HCV | CLIA test | Atellica IM Hepatitis C (aHCV) of the Siemens Healthineers commercial house | ≥ 1.0 |
| ***S.stercoralis* infection** | IVD *S.stercoralis* crude antigen | ELISA test | *SciMed*x® | ≥1.1 S/CO |
| ***Schistosoma* spp. infection** | Total antibodies anti-*Schistosoma* spp. | IHA test | *Fumouze*® | ≥1.0/80 |
| ***T.cruzi* infection** | Recombinant antigens (KMP11 and PFR2) | CMIA - *Arquitect* and an ELISA - *BioELISA* Chagas, *Biokit*® | *Abbot*® | ≥ 1.0 S/CO |
| CLIA: Chemiluminescence immunoassay, ELISA: Commercial Enzyme-Linked Immunosorbent Assay, HIV: Human immunodeficiency virus, anti-HBc: Hepatitis B core antigen, HBsAg: Australian antigen, anti-HCV: Hepatitis C total antibodies, IVD: In Vitro Diagnostic, KMP11: T. cruzi kinetoplastid membrane protein-11, PFR2: paraflagellar rod proteins 2, IHA: Indirect Hemagglutination. | | | | |

**Annex 3**. Case report form employed in the INPREMI intervention


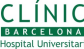


**Cuestionario T1 INPREMI**

Para todos los participantes: inmigrantes llegados los últimos 3 meses, inmigrantes llegados hace más de 3 meses, viajeros llegados en los 3 últimos meses.

*Las preguntas precedidas de * son de rellenado obligatorio*

**CODIFICACIÓN**

1. * Tipo participante: □ Viajero/VFR □ Inmigrante

*(Aviso: Los pacientes que se incluyan en el subestudio SEROLOGÍAS tienen que constar como INMIGRANTES)*

2. * Código de participante: _____________

3. * Código de centro: _____________

4. * Fecha de recogida de muestra: __ /__ / _____

5. * ¿A qué subestudio participa? (*seleccione una de las siguientes opciones*):

□ Serologías □ Multiresistencias □ Ambos

**ANTECEDENTES PERSONALES**

6. * Fecha de nacimiento: __ /__ / _____

7. * Sexo: □ Masculino □ Femenino

8. * Peso (en kg): ___________

9. * Talla (en cm): ___________

10. * ¿Se ha bañado en ríos o lagos durante su viaje/país de origen? □ Sí □ No

11. ¿Ha tenido contacto con animales durante su viaje/país de origen? □ Sí □ No

**Si tipo participante = inmigrante:**

12. * País origen: ___________ 13. * ¿De qué área proviene? (*seleccione una de las siguientes opciones*):

□ Urbana □ Rural □ Ambas

14. * Fecha de llegada a España: __ /__ / _____

Si solo conocen año: 01/07/AAAA, si también conocen mes: 15/MM/AAA)

15. * Medio de transporte (llegada) (*respuesta múltiple posible*):

□ Tierra □ Mar □Aéreo

16. * Número de países transitados durante el proceso migratorio: ___

17. * Nivel de estudios (*seleccione una de las siguientes opciones*):

□ Analfabeto □ Primaria □ Secundario □ Universitario □ Posgrado/doc

□ Estudiando □ Paro □ Otros. Especificar: ______________

18. * Profesión (*seleccione una de las siguientes opciones*):

□ Agricultura □ Tercer sector Industria

19. * Episodio de violencia: □ Sí □ No

19.1.* En caso afirmativo, tipo episodio violencia (*respuesta múltiple posible*) :

□ Victimas trata □ Violencia sexual □ Violencia física □ Otros. Especificar: _______

20. * ¿Ha tenido relaciones sexuales de riesgo? □ Sí □ No

21. * ¿Ha padecido mutilación genital femenina?

□ Sí □ No □ La consulta no ha dado pie a realizar esta pregunta.

22. Número de convivientes en el domicilio actual: ____

**FACTORES DE RIESGO**

23. Drogas: □ Sí □ No 23.1.En caso afirmativo, tipo drogas (*respuesta múltiple posible*):

□ Cocaína □ Heroína □ Recreativas □ Otros. Especificar: ___________

24. Alcohol: □ Sí □ No 24.1.En caso afirmativo, tipo alcohol (*respuesta múltiple posible*):

□ Vino □ Cerveza □ Destiladas/espirituosas □ Otras fermentadas

25. Tabaco (*seleccione una de las siguientes opciones*):

□ No fumador □ 0-10 cigarros al día □ 11-20 cigarros al día

□ >20 cigarros al día □ Ex fumador

26. Tipo dieta (*respuesta múltiple posible*):

□ Come carne habitualmente (>3 días/semana)

□ Come verdura habitualmente (>3 días/semana)

□ Come fruta habitualmente (>3 días/semana)

□ Come legumbres habitualmente (3 días/semana)

□ Come platos grasas saturadas habitualmente (>3 días /semana)

□ Come pescado habitualmente (>3 días/semana)

□ Come huevos >3 días/semana

□ Lácteos >3 días/semana

□ Vegetariano/vegano

**ANTECEDENTES PATOLÓGICOS**

27. * ¿Padece alguna enfermedad crónica? □ Sí □ No 27.1.* En caso afirmativo, tipo enfermedad (*respuesta múltiple posible*):

□ DM

□ Neoplasia solida

□ Neoplasia hematológica

□ VIH

□ Enfermedad inflamatoria intestinal

□ Conectivopatía o enfermedad reumática

□ Enfermedad pulmonar crónica

□ Hepatopatía crónica

□ Nefropatía crónica

□ Otras. Especificar: ___________

28. * ¿Inmunosupresión? □ Sí □ No 28.1.* En caso afirmativo, tipo inmunosupresión (*respuesta múltiple posible*):

□ Trasplante

□ Neoplasia onco/hematológica

□ Enfermedad autoinmune

□ VIH/Inmunodeficiencia

29. * ¿Tiene algún tipo de síntomas? □ Sí □ No 29.1.* En caso afirmativo, tipo de síntomas (*respuesta múltiple posible*):

□ Respiratorio □ Cutáneos □ Digestivos □ Sistémicos □ Neurológicos

29.1.1. En caso síntomas digestivos, ¿Ha tenido diarrea? □ Sí □ No 29.1.1.1. En caso afirmativo, número deposiciones al día: ____

30. ¿Toma alguna medicación actualmente? □ Sí □ No 30.1.En caso afirmativo, tipo medicación (*respuesta múltiple posible*):

□ Antibióticos □ Corticoides □ Immunosupresores

□ IBP □ Estatinas □ Antihipertensivos □ Antidiarreicos

30.1.1. En caso antibióticos, tipo, motivo, fecha inicio (*respuesta múltiple posible*):

| **Tipo antibiótico** | **Motivo** | **Fecha inicio** |
| --- | --- | --- |
| □ Betalactámicos | □ Respiratoria □ Neurológica □ Osteoarticluar □ Digestiva □ ORL □ Cutánea |  |
| □ Cefalosporinas | □ Respiratoria □ Neurológica □ Osteoarticluar □ Digestiva □ ORL □ Cutánea |  |
| □ Fosfomicina | □ Respiratoria □ Neurológica □ Osteoarticluar □ Digestiva □ ORL □ Cutánea |  |
| □ Quinolonas | □ Respiratoria □ Neurológica □ Osteoarticluar □ Digestiva □ ORL □ Cutánea |  |
| □ Sulfamidas | □ Respiratoria □ Neurológica □ Osteoarticluar □ Digestiva □ ORL □ Cutánea |  |
| □Antipalúdicos | □ Respiratoria □ Neurológica □ Osteoarticluar □ Digestiva □ ORL □ Cutánea |  |
| □ Otro.  Especificar:________ | □ Respiratoria □ Neurológica □ Osteoarticluar □ Digestiva □ ORL □ Cutánea |  |

31. ¿Tiene antecedentes de tuberculosis? □ Sí □ No

**Si subestudio = multiresistencias o ambos:**

32. ¿Tiene animales? □ Sí □ No 32.1.En caso afirmativo, tipo animales (respuesta múltiple posible):

□ Animales de compañía □ Animales de granja □ Animales salvajes

33. ¿Toma productos de herboristería? □ Sí □ No 33.1.En caso afirmativo, tipo producto herboristería: ____________________

34. ¿Ha tomado antibióticos en el último año? □ Sí □ No 34.1.1. En caso afirmativo, tipo, motivo, fecha inicio/final (*respuesta múltiple posible*):

| **Tipo antibiótico** | **Motivo** | **Fecha**  **inicio** | **Fecha**  **final** |
| --- | --- | --- | --- |
| □ Betalactámicos | □ Respiratoria □ Neurológica □ Osteoarticluar □ Digestiva □ ORL □ Cutánea |  |  |
| □ Cefalosporinas | □ Respiratoria □ Neurológica □ Osteoarticluar □ Digestiva □ ORL □ Cutánea |  |  |
| □ Fosfomicina | □ Respiratoria □ Neurológica □ Osteoarticluar □ Digestiva □ ORL □ Cutánea |  |  |
| □ Quinolonas | □ Respiratoria □ Neurológica □ Osteoarticluar □ Digestiva □ ORL □ Cutánea |  |  |
| □ Sulfamidas | □ Respiratoria □ Neurológica □ Osteoarticluar □ Digestiva □ ORL □ Cutánea |  |  |
| □ Antipalúdicos | □ Respiratoria □ Neurológica □ Osteoarticluar □ Digestiva □ ORL □ Cutánea |  |  |
| □ Otro.  Especificar:_________ | □ Respiratoria □ Neurológica □ Osteoarticluar □ Digestiva □ ORL □ Cutánea |  |  |

35. ¿Ha tomado probióticos en el último año? □ Sí □ No 35.1.Fecha inicio/final de los probióticos: __/__/____ - __/__/_____

36. ¿Ha tenido ingresos hospitalarios en el último año? □ Sí □ No 36.1.Número ingresos hospitalarios en el último año: ____

36.2.Fecha inicio/final ingreso: __/__/____ - __/__/_____

37. ¿Ha tenido cirugías en el último año? □ Sí □ No 37.1.Número de cirugías en el último año: ____ 37.2.Fecha última cirugía: __/__/_____

38. Aspecto de las heces (clasificación de Bristol) (*respuesta múltiple posible*):

□ Tipo 1: Trozos duros separados, que pasan con dificultad. Estreñimiento importante.

□ Tipo 2: Como una salchicha compuesta de fragmentos. Ligero estreñimiento.

□ Tipo 3: Con forma de morcilla con grietas en la superficie. Normal.

□ Tipo 4: Como una salchicha o serpiente, lisa y blanda. Normal.

□ Tipo 5: Trozos de masa pastosa con bordes definidos. Falta de fibra.

□ Tipo 6: Fragmentos pastosos, con bordes irregulares. Ligera diarrea.

□ Tipo 7: Acuosa, sin pedazos sólidos, totalmente liquida. Diarrea importante.

**Annex 4.** STROBE Guidelines Checklist

|  | **Item No** | **Recommendation** | **Page  No** |
| --- | --- | --- | --- |
| **Title and abstract** | 1 | (*a*) Indicate the study’s design with a commonly used term in the title or the abstract | 1 |
|  |  | (*b*) Provide in the abstract an informative and balanced summary of what was done and what was found | 3-4 |
| **Introduction** | | | |
| Background/rationale | 2 | Explain the scientific background and rationale for the investigation being reported | 5-6 |
| Objectives | 3 | State specific objectives, including any prespecified hypotheses | 6 |
| **Methods** | | | |
| Study design | 4 | Present key elements of study design early in the paper | 6-7 |
| Setting | 5 | Describe the setting, locations, and relevant dates, including periods of recruitment, exposure, follow-up, and data collection | 6 |
| Participants | 6 | (*a*) Give the eligibility criteria, and the sources and methods of selection of participants | 6-7 |
| Variables | 7 | Clearly define all outcomes, exposures, predictors, potential confounders, and effect modifiers. Give diagnostic criteria, if applicable | 7-9 |
| Data sources/ measurement | 8* | For each variable of interest, give sources of data and details of methods of assessment (measurement). Describe comparability of assessment methods if there is more than one group | 7-9 |
| Bias | 9 | Describe any efforts to address potential sources of bias | 6-7 |
| Study size | 10 | Explain how the study size was arrived at | N/A |
| Quantitative variables | 11 | Explain how quantitative variables were handled in the analyses. If applicable, describe which groupings were chosen and why | 8-9 |
| Statistical methods | 12 | (*a*) Describe all statistical methods, including those used to control for confounding | 8-9 |
|  |  | (*b*) Describe any methods used to examine subgroups and interactions | 8-9 |
|  |  | (*c*) Explain how missing data were addressed | Tables 1-4 |
|  |  | (*d*) If applicable, describe analytical methods taking account of sampling strategy | N/A |
|  |  | (*e*) Describe any sensitivity analyses | N/A |
| **Results** | | | |
| Participants | 13* | (a) Report numbers of individuals at each stage of study—eg numbers potentially eligible, examined for eligibility, confirmed eligible, included in the study, completing follow-up, and analysed | 9,  Figure 1 |
|  |  | (b) Give reasons for non-participation at each stage | 9,  Figure 1 |
|  |  | (c) Consider use of a flow diagram | Figure 1 |
| Descriptive data | 14* | (a) Give characteristics of study participants (eg demographic, clinical, social) and information on exposures and potential confounders | 9-10,  Table 1 |
|  |  | (b) Indicate number of participants with missing data for each variable of interest | Tables 1-4 |
| Outcome data | 15* | Report numbers of outcome events or summary measures | 10-12,  Tables 2-4 |
| Main results | 16 | (*a*) Give unadjusted estimates and, if applicable, confounder-adjusted estimates and their precision (eg, 95% confidence interval). Make clear which confounders were adjusted for and why they were included | 12,  Table 4 |
|  |  | (*b*) Report category boundaries when continuous variables were categorized | 8 |
|  |  | (*c*) If relevant, consider translating estimates of relative risk into absolute risk for a meaningful time period | N/A |
| Other analyses | 17 | Report other analyses done—eg analyses of subgroups and interactions, and sensitivity analyses | N/A |
| **Discussion** | | | |
| Key results | 18 | Summarise key results with reference to study objectives | 12 |
| Limitations | 19 | Discuss limitations of the study, taking into account sources of potential bias or imprecision. Discuss both direction and magnitude of any potential bias | 17-18 |
| Interpretation | 20 | Give a cautious overall interpretation of results considering objectives, limitations, multiplicity of analyses, results from similar studies, and other relevant evidence | 12-18 |
| Generalisability | 21 | Discuss the generalisability (external validity) of the study results | 16-17 |
| **Other information** | | | |
| Funding | 22 | Give the source of funding and the role of the funders for the present study and, if applicable, for the original study on which the present article is based | 19 |
